# The peculiarities of Kawasaki disease at the extremes of age: Two case reports

**DOI:** 10.1097/MD.0000000000017595

**Published:** 2019-10-18

**Authors:** Cristina Oana Mărginean, Lorena Elena Meliţ, Maria Oana Mărginean

**Affiliations:** a Department of Paediatrics; b Department of Paediatric, University of Medicine, Pharmacy, Sciences and Technology of Târgu Mures, Gheorghe Marinescu Street No. 38, Târgu Mures 540136, Romania.

**Keywords:** child, extremes of age, infant, Kawasaki disease, vasculitis

## Abstract

**Rationale:**

Extremes of age is an important risk factor for the development of coronary arteries aneurysms (CAAs) associated to Kawasaki disease **(**KD) along with male gender, prolonged fever and a delay in diagnosis or treatment.

**Patient concerns:**

We report two cases of KD in the extremes of age, a 5-month-old male infant and a 9-year-old child in order to underline the features of this disorder outside the typical age range of 1 to 4 years. The 5-month-old male was admitted in our clinic for generalized polymorphous exanthema and fever for approximately 7 days. The laboratory test pointed out leukocytosis and increased inflammatory biomarkers. The 9-year-old male child was admitted in our clinic for fever and submandibular adenopathy. The onset was approximately 5 days before the admission with a sudden development of unilateral, painless, submandibular lymphadenopathy for which the ENT specialist recommended antibiotics and nonsteroid anti-inflammatory drugs. In the 2nd day of admission, he presented severe desquamation of hands and soles.

**Diagnosis:**

Both cases were diagnosed with KD. The echocardiography showed no cardiac impairment in the infant, while in the older patient it revealed mild dilation of the left coronary artery.

**Interventions:**

Both patients received intravenously immunoglobulin and pulsed methylprednisolone.

**Outcomes:**

The evolution was favorable in both cases, but in the infant, the C-reactive protein levels persisted mildly elevated for approximately 2 months after the diagnosis.

**Lessons:**

The peculiarities of KD in the extremes of age are related to a higher frequency of incomplete features and an increased incidence of coronary artery lesions resulting in a delay of the diagnosis, and subsequent poorer outcomes.

## 1 Introduction

Kawasaki disease (KD) is an acute, systemic, self-limiting vasculitis of unknown etiology that was described for the first time in 1967 by Tomisaku Kawasaki.^[1]^ Common KD is defined by fever for more than 5 days associated with at least four of the following signs or symptoms: bilateral non-exudative conjunctival injection, erythema of the lips and oral mucosa, polymorphous skin rash, desquamation skin in the extremities, and/or unilateral painless cervical lymphadenopathy.^[2]^ There were also described atypical or incomplete forms of KD that do not fulfill all the clinical criteria mentioned above, but the presence of fever is mandatory.^[3]^ Cardiac impairment was reported to be up to 25% in untreated children and between 2% and 4% in those properly treated.^[4]^ KD is most frequently diagnosed in children with the age below 5 years with a slight predominance in males, male to female ratio 1.5:1.^[5]^ Even though over 90% of cases are diagnosed in infants and young children, the patients diagnosed outside the common age range may present worse outcome caused by a delay in diagnosis.^[3]^

Coronary arteries aneurysms (CAAs) are the most important complications during the subacute to convalescent phase.^[6]^ The recent guidelines recommend as standard treatment intravenous immunoglobulin (IVIG) and oral aspirin.^[7]^ The glucocorticoid therapy can be used as preemptive therapy associated to IVIG for predicted nonresponders.^[8]^

We report two cases of KD in the extremes of age, outside the age range of 1 to 5 years in order to underline the peculiarities related to the clinical symptoms, diagnosis and management in these ages.

The informed written consent was obtained from both patients’ mothers prior to the publication of these case reports.

## 2 Case reports

### 2.1 Case 1

**Figure 1:**
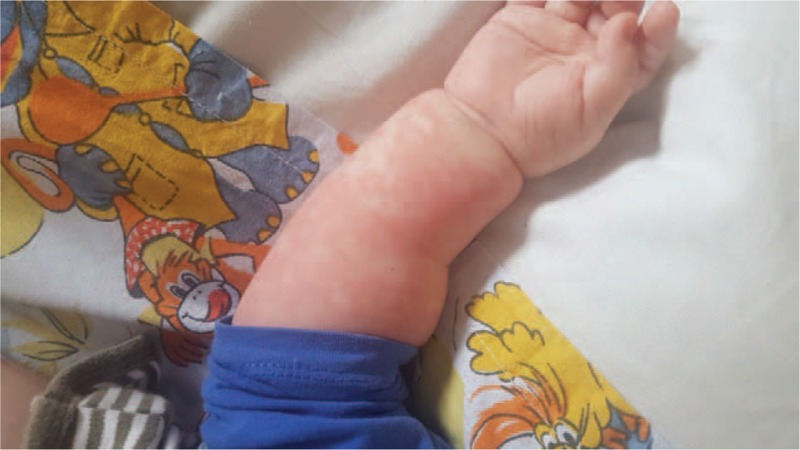
Aspect of the polymorphous exanthema in the infant.

The 1st case describes a 5-month-old male infant admitted for fever, generalized polymorphous exanthema, hyperemic conjunctivae, and diarrhea. The onset of the disease was approximately 2 weeks before the admission with a generalized polymorphous exanthema for which he received antihistaminic treatment, but after 7 days he associated fever, hyperemic conjunctivae, and diarrhea being admitted in the regional hospital. The fever persisted despite the antibiotic and symptomatic treatment, and therefore he was transferred in our clinic. *The clinical exam at the time of admission* revealed influenced general status, generalized polymorphous exanthema (Fig. 1), hyperemic pharynx, bilateral conjunctivitis, and approximately five diarrheic stools per day. *The laboratory tests* revealed leukocytosis (27,050/μL), with neutrophilia (19,900/μL), anemia (Hb 6.9 g/dL, Htc 19.7%, MEV 68.6 fL, MEH 24 pg), thrombocytosis (1,111,000/μL), elevated inflammatory biomarkers (CRP 62.01 mg/L, ESR 36 mm/h), hypoalbuminemia (2.8 g/dL), and mild hyponatremia (Na 135 mmol/L). The cultures performed from stools, blood and urine were all negative. The abdominal ultrasound revealed severe bloating. The chest X-ray and ENT exam did not reveal any pathological elements. The serological test for Toxoplasmosis, Rubella, Herpes virus, Cytomegalovirus, Epstein-Barr virus, and viral hepatitis were negative. The echocardiography was within normal ranges. We established *the diagnosis of KD* and we initiated IVIG, pulsed methylprednisolone and aspirin, we continued the antibiotic treatment (3rd generation cephalosporin), and we administered blood transfusion and substitutive treatment with human albumin by vein and electrolytes. The patient's evolution was slowly favorable, with intermittent fever within the 1st week after the IVIG and pulsed methylprednisolone, being discharged after approximately 3 weeks with the recommendation to continue the steroids orally for another 3 weeks. The CRP level remained mildly elevated for approximately 2 months from the discharge moment. Echocardiographic follow-up remained normal.

### 2.2 Case 2

**Figure 2:**
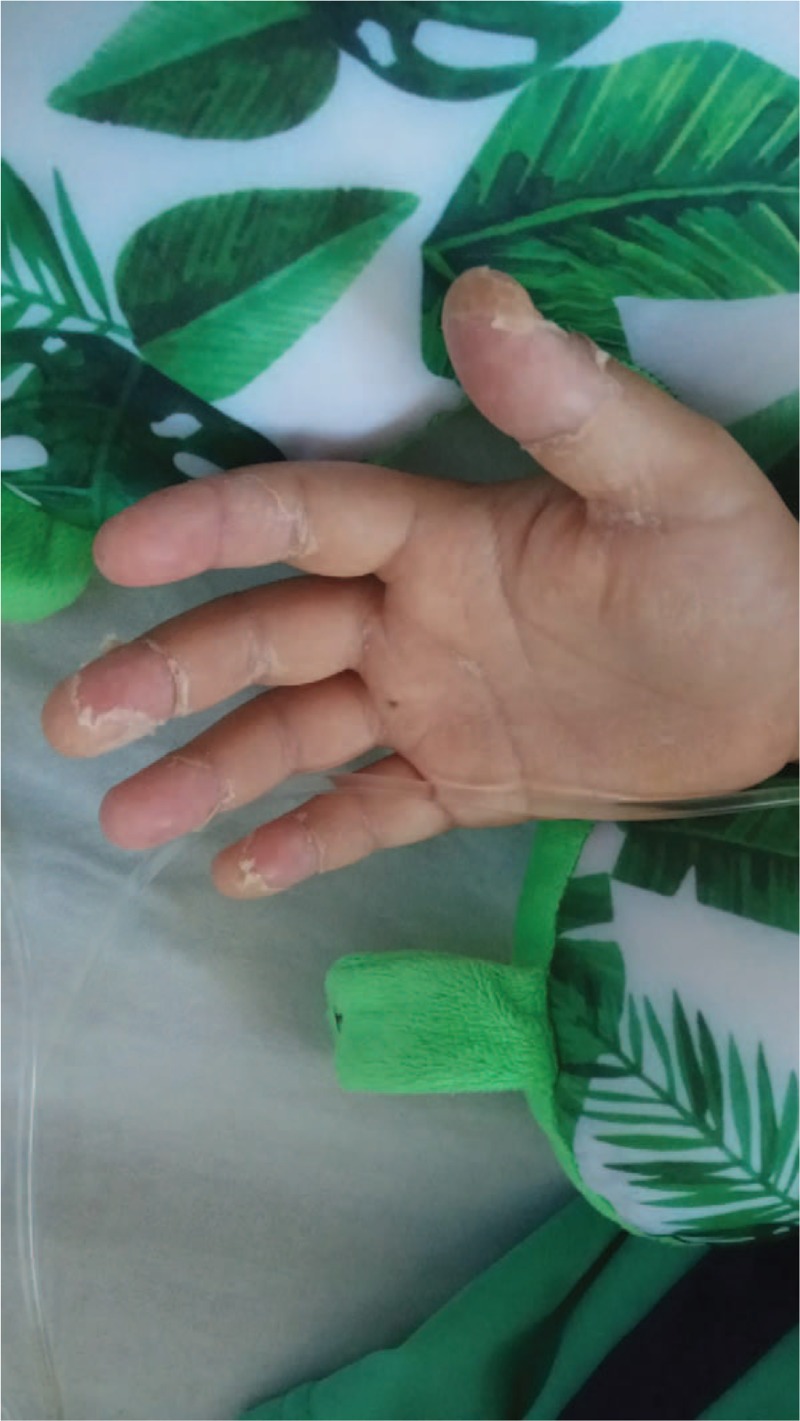
Aspect of skin desquamation in hands (9-year-old patient).

**Figure 3:**
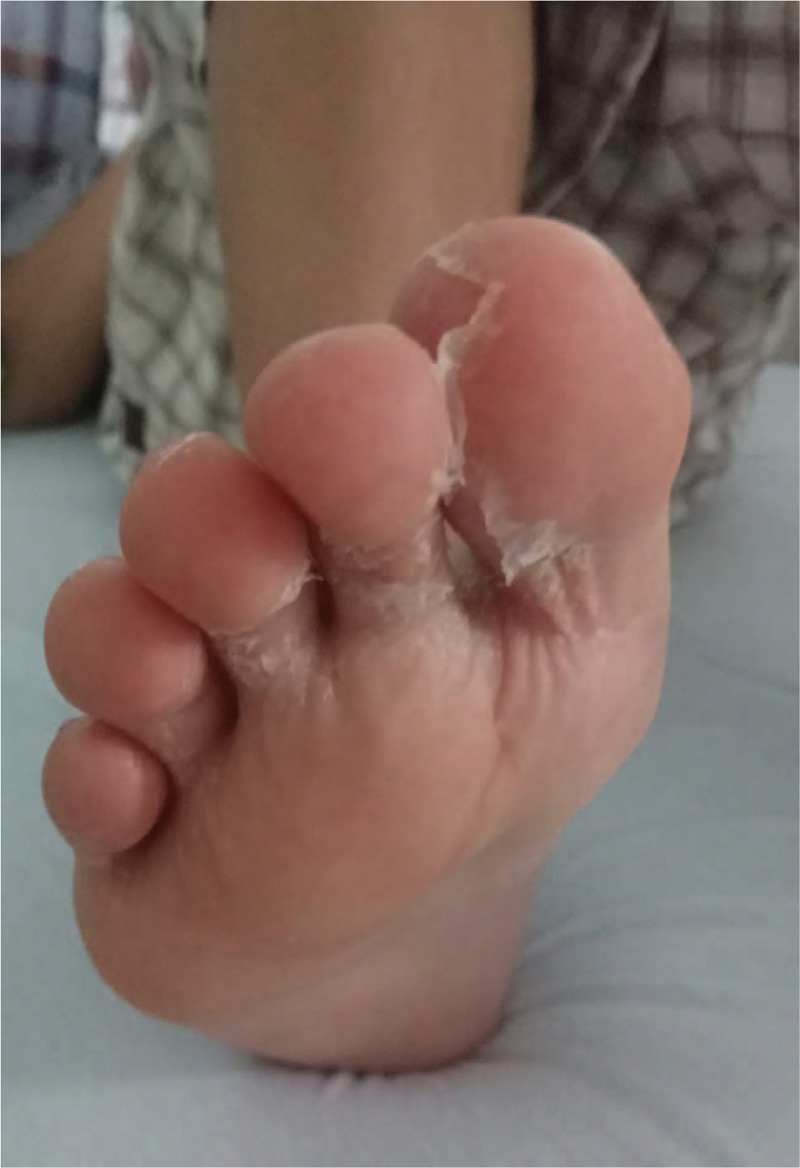
Aspect of skin desquamation in soles (9-year-old patient).

The 2nd case describes a 9-year-old male child admitted in our clinic for fever and submandibular adenopathy. The onset of the disease was 1 week before the admission with the development of a sudden right submandibular adenopathy for which the ENT specialist recommended antibiotic and nonsteroid anti-inflammatory drugs, but 3 days before the admission he associated fever, and he did not tolerate the antibiotic orally, thus being admitted in our clinic. *The clinical exam at the time of admission* revealed influenced general status, desquamation skin in hands and soles (Figs. 2 and 3), right submandibular adenopathy, and scratching lesions on the inferior limbs. The laboratory tests showed mild leukocytosis (12,330/μL) with neutrophilia (9680/μL), and increased inflammatory biomarkers (CRP 52.46 mg/L, ESR 29 mm/h). The serology for Toxoplasmosis, Rubella, Herpes virus, Cytomegalovirus, Epstein-Barr virus, and viral hepatitis were negative. The chest X-ray did not reveal any pathological elements. The abdominal ultrasound pointed out mild hepatomegaly. In the 2nd day of admission, the desquamation skin in extremities worsened, being associated with the persistence of fever, and therefore we raised the suspicion of KD. The echocardiography showed mild dilation of the left coronary artery, approximately 4 mm diameter. We established the diagnosis of KD with cardiac impairment and we initiated IVIG, pulsed methylprednisolone and aspirin, but also antipyretics. The patient's evolution was outstandingly favorable with the normalization of the CRP value after approximately 2 weeks. The echocardiographic follow-up after 1 week showed a decreasing diameter of the left coronary artery (3.16 mm).

## 3 Discussion

Extremes of age represents an important risk factor for the development of CAAs associated to KD along with male gender, prolonged fever and a delay in diagnosis or treatment.^[7]^ Thus, most often KD is misdiagnosed as infectious pathology, autoimmune disorders,^[9,10]^ malignancies,^[11,12]^ or even sepsis.^[13]^ Echocardiography is therefore essential in diagnosing different cardiopathies.^[14]^ The typical age range for KD is defined between 6 months and 4 years, being reported that 15% of the cases occur outside this range.^[15]^ Our cases are both outside the specific age range for KD, a 5-month-old infant and a 9-year-old child, both males strengthening the reports that male gender is more predisposed to develop this disorder.^[5]^ Even though the etiology remains unknown, well-designed national screening programs similar to those used for other rare disorders^[16]^ could be useful on long-term to identify the risk factors for developing KD. Several criteria are mandatory for diagnosing typical KD.^[2]^ Therefore, physician's communications skills are essential for a thorough anamnesis.^[17]^ Fever, one of the most important clinical criteria, was present in both our cases, even though the infant was febrile for a greater amount of time in comparison to the older patient. Besides fever, the infant also associated bilateral conjunctival injection, polymorphous skin rash, and hyperemic pharynx. On the other hand, the 9-year-old patient presented also unilateral painless lymphadenopathy, desquamation skin in the extremities and mild dilation of the left coronary artery, while the echocardiography of the infant was normal. Based on all these findings, we may state that both our cases can be defined as incomplete KD. Similar to other conditions,^[18–21]^ systemic inflammation is a well-documented feature of KD that increases the risk of CAAs. Among the common findings in KD patients were reported: leukocytosis with neutrophilia, normocytic normochromic anemia, thrombocytosis, or elevated inflammatory biomarkers.^[22]^ Both our patients presented leukocytosis with neutrophilia and elevated inflammatory biomarkers, though more expressed in our infant. Moreover, severe thrombocytosis and anemia requiring blood transfusion were encountered only in the younger patient. Hyponatremia is another laboratory finding commonly encountered in patients with KD that was associated with the development of CAAs.^[22]^ Contrariwise, our cases failed to prove this relationship because mild hyponatremia was found only in the infant, while the echocardiography showed coronary changes in the older patient. Besides hyponatremia, another marker is hypoalbuminemia that might be related to adverse coronary outcomes.^[3]^ Contrariwise, even though our infant presented hypoalbuminemia, he did not associate coronary injuries, while in the older patient the level of albumin was normal. Nevertheless, laboratory parameters remain unspecific and they might suggest also other conditions.^[23,24]^

Several studies focused on assessing the features of KD according to the patient's age. Therefore, a study performed on 185 children, with the age ranging between 1 month and 10 years, including 22 cases <5 months of age, 131 cases between 6 months and 4 years and 32 cases >5 years of age, stated that the clinical and laboratory findings do not vary significantly with the age.^[25]^ Contrariwise, other studies performed on KD patients at the extremes of age suggested that infants below the age of 6 months were more frequently encountered with giant aneurysms, incomplete KD or atypical symptoms.^[26]^ Our 5-month-old patient also expressed incomplete KD and diarrheic stools as atypical feature, but without coronary impairment. On the contrary, older children with KD were reported to be predominantly males, with a higher incidence of cervical lymphadenopathy and coronary abnormalities, and to complain of abdominal and joint pain.^[15,27,28]^ Similarly to these reports, the 9-year-old patient was also a male, presented the onset with unilateral painless cervical lymphadenopathy, and he was also found with left coronary artery dilation. Another large study, performed on 1374 patients, proved that extremes of age are more predisposed to present with less than 4 of the classic KD criteria.^[29]^ Similarly, both our patients were diagnosed with incomplete KD. The same authors stated that patients diagnosed before the age of 6 months are more likely to present lower albumin and hemoglobin levels, higher platelet counts and increased incidence of coronary lesions in comparison to children above the age of 9 years whose laboratory profiles were more favorable.^[29]^ Similarly, our younger patient presented with all the findings mentioned above except for coronary lesions in comparison to the older one who had only a mild leukocytosis with neutrophilia, but in exchange, he was found with coronary changes.

According to the guidelines of the Japanese Society of Pediatric Cardiology and Cardiac Surgery, prednisolone or intravenous methylprednisolone can be included in the first-line treatment of KD along with IVIG for predicted nonresponders.^[30]^ Moreover, the revised guidelines of the American Heart Association suggested that a longer tapering regimen of glucocorticoids may also be taken into account for this group of patients.^[31]^ Based on their clinical features and laboratory findings, both our patients were considered to be IVIG predicted nonresponders and therefore, we initiated IVIG and intravenous methylprednisolone as a first-line therapy in both patients followed by oral tapered courses of prednisolone with favorable outcome in both cases.

## 4 Conclusions

KD is a challenging entity for pediatricians, but in the extremes of age is even more difficult to be diagnosed due to both its scarce incidence and the pediatrician's reluctance. The peculiarities of KD in the extremes of age are related to poorer outcomes.

## Acknowledgments

The authors express their gratitude to Dr. Man Lidia who contributed to the management of the second case.

## Author contributions

**Conceptualization:** Cristina Oana Mărginean, Lorena Elena Meliţ, Maria Oana Mărginean.

**Data curation:** Cristina Oana Mărginean, Lorena Elena Meliţ.

**Investigation:** Cristina Oana Mărginean, Lorena Elena Meliţ.

**Methodology:** Cristina Oana Mărginean.

**Supervision:** Cristina Oana Mărginean, Lorena Elena Meliţ.

**Validation:** Cristina Oana Mărginean, Lorena Elena Meliţ, Maria Oana Mărginean.

**Visualization:** Lorena Elena Meliţ.

**Writing – original draft:** Cristina Oana Mărginean, Lorena Elena Meliţ, Maria Oana Mărginean.

**Writing – review & editing:** Cristina Oana Mărginean, Lorena Elena Meliţ, Maria Oana Mărginean.
